# Clinical efficiency of mesenchymal stem cells for bone regeneration and osseointegration

**DOI:** 10.6026/973206300222949

**Published:** 2026-05-31

**Authors:** Ghazala Khurshid, Reddy Chaithanya, Akshyata Panda, Khushboo Changani, Shailesh Menat, Haren Pandya

**Affiliations:** 1 Department of Prosthodontics and Crown & Bridge, Consultant at Lake View Hospital, Ranchi, Jharkhand, India; 2 Department of Prosthodontics, Government Dental College and Hospital, Kadapa andhra Pradesh, India; 3 Department of Periodontics and Oral Implantology, Institute of Dental Sciences, Siksha O Anusandhan University, Bhubaneswar, India; 4 Department of Oral and Maxillofacial Surgery, Narsinhbhai Patel Dental College and Hospital, Sankalchand Patel University, Visnagar, Gujarat, India; 5 Department of Oral Surgery, DDU, NADIAD, Gujarat, India

**Keywords:** Mesenchymal Stem Cells (MSCs), bone regeneration, osseointegration, dental implants, bone grafting, implant stability, cone beam computer tomography (CBCT), tissue engineering, and regenerative dentistry

## Abstract

Achieving predictable bone regeneration and optimal osseointegration remains a clinical challenge in dental implant therapy, particularly in sites with compromised bone quality. Therefore, it is of interest to evaluate the clinical efficiency of mesenchymal stem cells (MSCs) in enhancing bone regeneration and improving osseointegration in patients receiving dental implants. Hence, sixty patients were randomly divided into two groups: Group A received MSCs combined with bone grafts, while Group B received conventional bone grafts alone. Over a six-month follow-up period, Group A demonstrated significantly higher implant stability quotient (ISQ) values, greater bone volume gain and increased bone density compared to Group B. Implant survival was 100% in the MSC group, whereas the control group showed a survival rate of 96.7%. Thus, we show that the adjunctive use of mesenchymal stem cells can improve clinical outcomes in oral implantology.

## Background:

Dental implantology is a cornerstone of oral rehabilitation, providing functional and aesthetic restoration for partially or completely edentulous patients. Osseointegration, defined as the direct structural and functional connection between living bone and a load-bearing implant, is essential for long-term implant success [1]. Achieving predictable osseointegration remains challenging in conditions with compromised bone quality or quantity, including post-extraction defects, periodontal disease and trauma. Conventional bone regeneration techniques include autografts, allografts and synthetic grafting materials. Although effective, these approaches present limitations such as donor site morbidity, immunogenic reactions and limited regenerative capacity [2, 3]. Advances in tissue engineering and regenerative medicine have introduced biological alternatives aimed at overcoming these limitations. Among these, mesenchymal stem cells (MSCs) have gained attention for their regenerative potential in implant dentistry. MSCs are multipotent progenitor cells capable of differentiating into osteoblasts, chondrocytes and adipocytes. They also promote angiogenesis and osteogenesis, enhancing bone formation *in vivo* [4]. Additionally, MSCs exert paracrine effects that support wound healing and modulate inflammatory responses [5]. MSCs derived from bone marrow, adipose tissue and dental tissues have been studied for maxillofacial bone regeneration in both preclinical and clinical models [6]. Recent studies suggest that MSC-based therapies may improve implant stability, bone density and healing outcomes when used during dental implant placement [7, 8]. Despite encouraging findings, evidence supporting the routine clinical application of MSCs in oral implantology remains limited. Further well-designed human clinical trials are required to establish their efficacy and translational relevance. Therefore, it is of interest to evaluate the clinical efficiency of mesenchymal stem cells in enhancing bone regeneration and osseointegration in patients undergoing dental implant therapy.

## Methodology:

After receiving approval from the Institutional Ethical Committee (IEC number: Pr.011/IEC/GDCH/2024-25/06), this prospective, randomised controlled clinical study was carried out and all participants provided written informed consent. The goal was to evaluate the clinical effectiveness of mesenchymal stem cells (MSCs) in encouraging osseointegration and bone regeneration in patients having dental implants placed. 60 patients in need of dental implants in regions with inadequate bone volume were recruited and divided into two groups at random: While Group B (n=30) underwent traditional implant placement with standard bone grafting techniques alone, Group A (n=30) received MSC therapy in addition to implant placement. Predetermined inclusion criteria were used to choose the participants, including age between 20 and 60, good oral and general health and the existence of bone defects that need to be filled in. Patients who were immunocompromised, smoked, heavily drank alcohol, were pregnant or nursing, or had systemic diseases like uncontrolled diabetes were not included. In Group A, bone marrow aspiration was used to extract mesenchymal stem cells from the iliac crest of the patients while they were under local anaesthesia. MSCs were separated from the aspirate using density gradient centrifugation and they were subsequently applied to the implant site after being suspended in sterile saline or a biocompatible scaffold. In Group B, traditional graft materials devoid of MSCs were used for bone augmentation. Every implant was positioned in accordance with a standard operating procedure. Intraoperative measurements of primary implant stability were made using resonance frequency and insertion torque values. Analysis (RFA) to determine scores for the implant stability quotient (ISQ). At one week, one month, three months and six months following surgery, postoperative evaluations were performed. At every visit, clinical outcomes such as infection, inflammation and soft tissue healing were tracked. Cone-beam computed tomography (CBCT) radiographic evaluations were conducted at baseline and six months after surgery to assess osseointegration and bone regeneration. During secondary procedures, histomorphometric analysis was carried out when clinically indicated. SPSS version was used to analyse all of the data. ANOVA or the Student's t-test where used to compare continuous variables like bone density and ISQ scores and the Chi-square test was used to evaluate categorical data. Statistical significance was defined as a p-value of less than 0.05.

## Results:

In this study, 60 patients requiring dental implants with bone augmentation were randomly divided into two groups: Group A (n=30) received mesenchymal stem cells in conjunction with bone grafts, while Group B (n=30) received conventional grafting materials alone. Patients were followed over a six-month period and evaluated clinically and radiographically for implant stability and bone regeneration. Group A showed significantly higher ISQ values at 6 months compared to Group B, indicating improved osseointegration. Mean ISQ increased from 63.5±4.2 to 75.2±3.6 in Group A, versus 61.8±5.0 to 70.4±4.1 in Group B (p < 0.01). CBCT analysis at 6 months showed greater bone volume and density in the MSC group. Mean bone fill in Group A was 215.6 mm^3^ compared to 178.3 mm^3^ in Group B (p < 0.001) and bone density was significantly higher in Group A (879 HU) than in Group B (725 HU) (p< 0.01). Healing was uneventful in both groups, with no serious complications reported. Implant survival was 100% in Group A and 96.7% in Group B (one failure, not statistically significant) (Table 1).

## Discussion:

The effects of mesenchymal stem cells (MSCs) on bone regeneration and implant osseointegration outcomes in patients undergoing oral implant placement were assessed in this clinical study. The results showed that the addition of MSCs significantly improved both implant stability and bone regenerative parameters when compared to conventional grafting techniques alone. The statistically significant increase in Implant Stability Quotient (ISQ) values in Group A (MSC group) over a six-month period indicates superior osseointegration, which is consistent with earlier findings that MSCs can promote osteogenic differentiation and improve the integration of biomaterials with host bone [9]. The mean ISQ difference between the two groups at the end of the study period suggests a biologically relevant improvement in secondary implant stability, which is crucial for long-term implant success. The CBCT radiographic evidence further confirmed MSCs' capacity for regeneration. The idea that MSCs speed up bone formation and mineralisation is supported by Group A's noticeably greater bone volume and density at six months. These results are in line with those of Kurmanalina *et al.* Who found that sites treated with stem cell-enhanced grafts had better trabecular structure and bone density [8]. A crucial sign of successful osseointegration, more mature and compact bone is reflected in the MSC group's higher HU values [4]. This better result most likely has a multifactorial underlying mechanism. In addition to differentiating into osteoblasts, MSCs also release paracrine factors like TGF-β and VEGF, which control bone remodelling and encourage neovascularisation [5]. The faster and better-organized bone regeneration seen in the MSC-treated group could be explained by these effects. The lack of serious postoperative problems in either group further suggests that MSCs' anti-inflammatory and immunomodulatory qualities may promote improved healing and decreased complication rates. While implant survival rates were high in both groups, the 100% success rate in the MSC group suggests a potential for improved clinical predictability, particularly in compromised bone situations. However, the small sample size and short follow-up duration limit the ability to draw long-term conclusions. Histomorphometric analysis, though limited to secondary surgeries, supported the radiographic findings, showing more mature bone formation in the MSC-treated sites. These findings are in line with emerging literature that supports the integration of stem cell therapy into routine regenerative dental protocols [9, 10]. Overall, this study offers encouraging proof of MSCs' clinical effectiveness in improving bone regeneration and osseointegration in oral implantology. To validate these findings and establish standardised procedures for MSC harvesting, processing and application, more studies with bigger cohorts and longer follow-up times are required. Clinical evidence suggests that mesenchymal stem cells enhance bone regeneration around dental. Clinical evidence suggests that mesenchymal stem cells enhance bone regeneration around dental implants by promoting new bone formation at the peri-implant site [11]. MSCs stimulate osteogenesis and angiogenesis, thereby improving the biological processes required for successful osseointegration [12]. Recent systematic reviews have reported increased bone density and higher implant stability values when MSC-assisted regenerative approaches are used compared with conventional grafting methods [13]. Clinical trials conducted in human subjects have demonstrated superior radiographic bone outcomes and improved healing patterns with the adjunctive use of MSCs during implant placement [14]. However, despite these promising results, large-scale randomized controlled trials are still required to establish their routine clinical applicability in oral implantology [15]. Yamada & Egusa (2018) conducted a systematic review and meta-analysis to evaluate the effectiveness of mesenchymal stem cells (MSCs) in maxillary bone regeneration for dental implant rehabilitation. The study found that the use of MSCs may enhance bone formation and produce viable, vascularized bone tissue. However, the results were not always statistically significant compared with conventional methods. Overall, MSC-based bone regeneration shows promising potential as a minimally invasive alternative to traditional autologous bone grafting [16].

## Conclusion:

We show that mesenchymal stem cells (MSCs) significantly enhance osseointegration and bone regeneration in dental implant procedures. Patients receiving MSC-enhanced grafts showed superior implant stability, bone volume and density compared to conventional grafting. Further large-scale studies are needed to confirm long-term benefits to standardize clinical protocols.

## Figures and Tables

**Table 1:** Comparative results between Group A (MSC Group) and Group B (Control Group). Clinical and radiographic outcomes at 6 months post-implant placement

**Parameter**	**Group A (MSC group)**	**Group B (Control group)**	**p-value**
Sample size (n)	30	30	-
Initial ISQ (mean±SD)	63.5±4.2	61.8±5.0	0.12
Final ISQ at 6 months (mean±SD)	75.2±3.6	70.4±4.1	<0.01
Bone fill (mm^3^)	215.6±18.4	178.3±21.7	<0.001
Bone density (Hounsfield Units)	879±55	725±67	<0.01
Implant survival (%)	100%	96.70%	0.31
Complication rate	0%	3.3% (1 case)	NS
Note: ISQ = Implant
Stability Quotient;
NS = Not Significant.
